# Development of Improved Fruit, Vegetable, and Ornamental Crops Using the CRISPR/Cas9 Genome Editing Technique

**DOI:** 10.3390/plants8120601

**Published:** 2019-12-13

**Authors:** Lígia Erpen-Dalla Corte, Lamiaa M. Mahmoud, Tatiana S. Moraes, Zhonglin Mou, Jude W. Grosser, Manjul Dutt

**Affiliations:** 1 Department of Agronomy, Universidade Estadual de Londrina, Londrina 86057-970, PR, Brazil; ligiacorte@uel.br; 2 Pomology Department, Faculty of Agriculture, Mansoura University, 35516 Mansoura, Egypt; lamiaa.mahmoud@ufl.edu; 3 Citrus Research and Education Center, University of Florida, Lake Alfred, FL 33850, USA; jgrosser@ufl.edu; 4 Centro de Energia Nuclear na Agricultura, Universidade de São Paulo, Piracicaba 13416-000, SP, Brazil; t.moraes@usp.br; 5 Department of Microbiology and Cell Sciences, University of Florida, Gainesville, FL 32603, USA; zhlmou@ufl.edu

**Keywords:** CRISPR/Cas9, genome editing, gene knockout, horticultural plants, precision editing

## Abstract

Horticultural crops, including fruit, vegetable, and ornamental plants are an important component of the agriculture production systems and play an important role in sustaining human life. With a steady growth in the world’s population and the consequent need for more food, sustainable and increased fruit and vegetable crop production is a major challenge to guarantee future food security. Although conventional breeding techniques have significantly contributed to the development of important varieties, new approaches are required to further improve horticultural crop production. Clustered regularly interspaced short palindromic repeats (CRISPR)/CRISPR-associated protein 9 (Cas9) has emerged as a valuable genome-editing tool able to change DNA sequences at precisely chosen loci. The CRISPR/Cas9 system was developed based on the bacterial adaptive immune system and comprises of an endonuclease guided by one or more single-guide RNAs to generate double-strand breaks. These breaks can then be repaired by the natural cellular repair mechanisms, during which genetic mutations are introduced. In a short time, the CRISPR/Cas9 system has become a popular genome-editing technique, with numerous examples of gene mutation and transcriptional regulation control in both model and crop plants. In this review, various aspects of the CRISPR/Cas9 system are explored, including a general presentation of the function of the CRISPR/Cas9 system in bacteria and its practical application as a biotechnological tool for editing plant genomes, particularly in horticultural crops.

## 1. Introduction to Genome Editing

Historically, genetic modifications at the DNA level have resulted in the development of improved plant varieties. Naturally occurring genetic modifications such as random mutations have given rise to most of the ancestors of today’s cultivated plants. Induced modifications have improved cultivars by applying mutagenesis approaches using radiation or chemical agents [1,2]. These mutagenesis approaches randomly induce multiple double-strand breaks (DSBs) in the plant’s genome, and the natural DNA repair machinery tries to repair the damaging modifications. These repair mechanisms occur naturally and have evolved over time as an essential component for the survival of all species. As these methods randomly introduce mutations into the genome, they have unpredictable results, require screening of many individuals, and can rarely lead to a desirable phenotype [3,4].

Genome editing using engineered nucleases can precisely delete, replace, or insert specific sequences in a targeted location of the organism’s genome for the generation of novel traits. The application of genome editing requires synthetic nucleases that introduce DSBs into a definite region of the genome and the natural DNA repair machinery that makes the modifications. The capacity to generate specific damage at the DNA level and the repair responses constitute the fundamental principles behind gene editing [5,6]. The zinc finger nucleases (ZFNs) were originally used for genome editing, followed by transcription activator-like effector nucleases (TALENs). ZFNs are a combination of the DNA-binding zinc-finger motifs with a domain of the *Fok*I endonuclease [7]. Transcription activator-like effector (TALE) proteins are DNA-binding domains isolated from different plant bacterial pathogens of the genus *Xanthomonas*. These proteins are secreted into the host plant cell during infection. TALENs consist of a TALE DNA-binding domain in combination with the *Fok*I endonuclease and have been preferred over ZFNs for years [8,9]. Each has advantages and disadvantages, but both are expensive and laborious to engineer, which has prevented them from being widely used [10,11,12].

Since 2013, an efficient genome-editing tool has been developed. This tool is based on the bacterial clustered regularly interspaced short palindromic repeats (CRISPR)/CRISPR-associated protein 9 (Cas9) system. This system is a constituent of the bacteria’s adaptive immune system [13,14]. The major elements of the system are small non-coding RNAs that guide a complex of multiple Cas proteins or a single large Cas protein to introduce DSBs into a specific DNA sequence. Thus far, CRISPR/Cas9 is the most extensively used genome-editing system. The ability to generate mutations with ZFNs, TALENs, and CRISPR/Cas9 is very similar [10]. However, in a short time, CRISPR/Cas9 has received widespread attention, and there are numerous examples of gene mutation and transcriptional regulation control reported in both model and crop plants [15,16,17,18], highlighting the tool’s clear advantages of simplicity, accessibility, low cost, and versatility.

In this review, several aspects of the CRISPR/Cas9 system are explored, including a general presentation of the CRISPR/Cas9 system in bacteria, its practical application as a biotechnological tool for editing plant genomes, and major achievements in horticultural crops.

## 2. Mechanism and Application of the CRISPR/Cas9 System for Genome Editing in Plants

CRISPR/Cas9 was identified as part of the immune system in bacteria and archaea, which protects against invading phages and plasma DNA [19]. In prokaryotic organisms, tandem repeats were observed to be regularly interspaced by non-repetitive sequences, collectively referred to as CRISPR [20]. These 29-nucleotide (nt) repeat sequences separated by various 32-nt spacer sequences were first identified in the *Escherichia coli* genome in 1987 [21]. Later, similar sequences were detected in other groups of bacteria and archaea [22]. The CRISPR spacer sequences have been observed to be highly homologous with exogenous sequences from invading viruses, phages, and plasmids. The bacteria can introduce parts of these exogenous DNA into their own genomes, which is subsequently transcribed to form two small RNAs, CRISPR RNA (“crRNA”) and transactivating CRISPR RNA (“tracrRNA”). The tracrRNA and crRNA act to identify invading foreign nucleic acids and to guide a Cas endonuclease to eliminate them through specific cleavage [23,24]. Whenever there is an invasion of foreign organisms, the immune system uses the information on the spacer DNA and the Cas nuclease to protect the cell [25].

**Figure 1:**
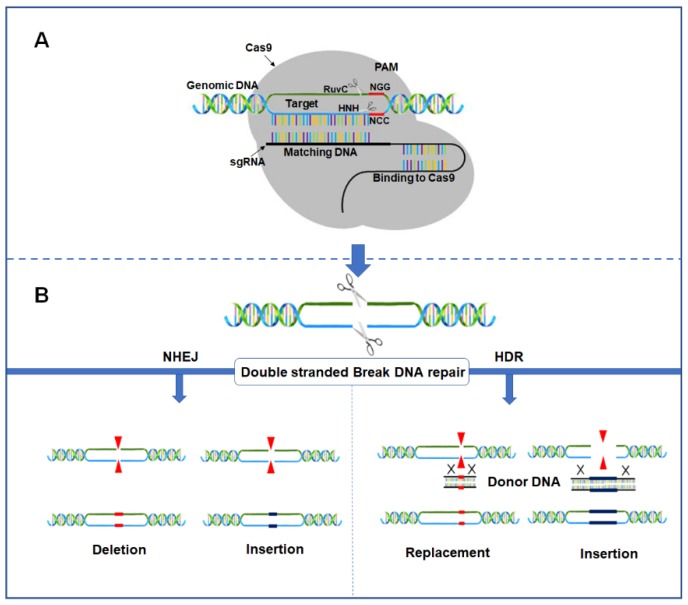
Schematic diagram of CRISPR/Cas9-mediated genome editing. (**A**) Representation of the Cas9/sgRNA/target DNA complex. The sgRNA is represented by the black line. The target sequence is indicated by the blue strand of genomic DNA. The protospacer adjacent motif (PAM) sequence is highlighted in red. The enzyme Cas9 is represented by the gray background. The catalytic domains RuvC and HNH responsible for non-complementary and complementary strand break, respectively, are indicated by scissors. (**B**) Repair of the DNA double-strand breaks. Two mechanisms may be involved in the repair: (i) the non-homologous end joining (NHEJ) or (ii) the homology-directed repair (HDR).

The CRISPR/Cas9 genome-editing technology has been developed based on the *Streptococcus pyogenes* CRISPR/Cas9 system in which the Cas9 endonuclease binds crRNA and tracrRNA [26,27]. Artificially, these two naturally occurring small RNAs can be fused to form one molecule. This molecule is termed as the single-guide RNA (sgRNA) and is complementary to a specific part of a target gene (approximately 20 nucleotides) in a region that contains a protospacer adjacent motif (PAM) [22]. The sgRNA also contains a “scaffold” sequence necessary for Cas9 binding. Cas9 is guided by sgRNA and requires the PAM sequence to recognize the target site and differentiate between self and non-self-sequences (Figure 1A) [25]. In other words, the PAM sequence (5′-NGG-3′, where N = any nucleotide) is located downstream of the target site, and the Cas9 nuclease creates blunt-end DSBs between the 3rd and 4th nucleotides upstream of the PAM motif [23]. Lack of PAM prevents Cas9 from cleaving the target sequence [28].

Recently, a new Cas protein named Cpf1 (also known as Cas12a), identified in *Prevotella* and *Francisella*, has also been used as a nuclease in plant genome editing [29,30,31]. Unlike CRISPR/Cas9, the CRISPR/Cpf1 system requires a single crRNA that does not need to couple with a tracrRNA, allowing the use of a smaller gRNA. Cpf1 recognizes an alternative PAM sequence (5′-TTTN-3′) upstream of the target site, increasing the range of targets for forming DSBs. Additionally, unlike Cas9, Cpf1 generates staggered cuts with 5ʹ overhangs, analogous to traditional restriction enzyme cloning, that are 18–23 bp distal from the PAM sequence [31].

Expression of Cas9 with sgRNA leads to introduction of DSBs, which can be repaired via two major pathways: non-homologous end joining (NHEJ) and homology-directed repair (HDR; Figure 1B) [5]; DSB repair via NHEJ is the most frequent and is characterized by the fusion of the break ends where insertions or deletions (indels) of one or more random nucleotides can occur, leading to imperfect repair and mutations [32]. When DSBs are repaired by NHEJ, it typically results in gene knockout or loss of protein function. Thus, NHEJ can be used as an alternative to RNAi and other gene silencing techniques [33]. Alternatively, if an artificial repair template is provided, HDR is induced and larger exogenous DNA fragments can be inserted, or single nucleotides can be exchanged in a specified genomic location, resulting in a more precise gene editing. However, the target has a low mutation frequency since it is difficult to insert an artificial DNA repair template into the plant cells [34,35].

**Figure 2:**
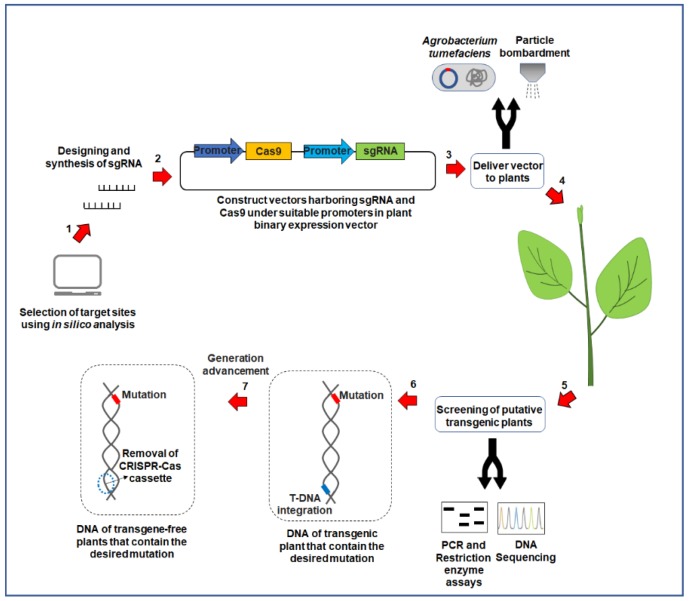
Overview of the basic flow of the CRISPR/Cas9 system in plant genome editing. 1. Select a genomic target where the mutation is to be introduced. 2. Design the sgRNA complementary to the expected target sequence. 3. The sgRNA and Cas9 under suitable promoters are cloned in plant binary expression vectors. 4. The components of the CRISPR/Cas9 system construct are delivered into the plants, via *Agrobacterium*-mediated transformation or particle bombardment. 5. The mutations in regenerated transgenic plants are identified using restriction enzyme assays and sequencing. 6 and 7. The removal of the CRISPR/Cas9 cassettes is possible in subsequent generations of plants.

In plants, efficient CRISPR/Cas9-based genome editing comprises of the following steps as depicted in Figure 2. Initially, it is necessary to choose a region in the gene of interest that is unique in the genome and located upstream of a 5′-NGG-3′ PAM sequence [13]. The sgRNA must have homology to the sequence of interest (approximately 20 bp sequence) and should also contain a “scaffold” sequence necessary for Cas9 binding [13,36]. When expressed, the Cas9 protein and sgRNA form a complex that can bind to any genomic sequence with a PAM but will only cleave the target if it is sufficiently homologous to the sgRNA [37]. sgRNAs are solely responsible for recruiting Cas9 to specific genomic loci, and their optimal design is critical for successful gene editing. It is also important to minimize off-target possibilities, i.e., when sgRNA matches additional sites that are similar to the target sequences, unwanted mutations could occur [37,38]. If the goal is to induce HDR repair mechanism, it is necessary to provide a DNA repair template containing the desired mutation in addition to the sgRNA(s) and the Cas9 nuclease. To facilitate this process, there must be an additional homologous sequence immediately upstream and downstream of the target (named left and right homology arms, respectively). The repair template can be delivered as single or double-stranded oligonucleotide as well as a double-stranded DNA plasmid [39,40]. Then, the Cas9 and sgRNA expression cassettes are incorporated into the plant’s genome, normally via *Agrobacterium*-mediated transformation [41,42]. Expression of Cas9 and sgRNA introduces DSBs at the target site [43]. After being repaired via NHEJ or HDR, desired mutations in regenerated transgenic plants are identified by restriction enzyme assays or sequencing [44]. Usually, the transgenic population contains a wide variety of mutations or edits within the target gene. Often, the following four genotypes are generated in T0 lines: homozygote (same mutation in two alleles), heterozygote (mutation of a single allele), biallelic (different mutations in two alleles) and chimera (more than two different mutations) [45]. Some plants remain unmodified because of the absence of Cas9/sgRNA expression or insufficient target cleavage. Even when a DNA repair template is provided to induce repair via HDR, some of the DSBs are still repaired by NHEJ, leading to undesirable mutations [46]. Since the Cas9/sgRNA transgene and the target region are often at different locations in the genome, removal of the Cas9/sgRNA transgene through segregation is possible in the subsequent generations.

Thus far, the majority of published studies with the CRISPR/Cas9 system involve NHEJ-mediated single-gene knockout or down-regulation [15,16,17,18]. However, when Cas9 is combined with multiple sgRNAs specific for different target genes [47], NHEJ may also introduce targeted chromosome deletion [42] and knock out the whole gene family, which provides the possibility to introduce multiple traits in an elite variety [48,49,50]. Additionally, HDR can be employed to generate a specific sequence change for gene correction, gene replacement, and gene knock-in [51,52].

## 3. Genome Editing in Fruit Crops

In plants, genome editing using the CRISPR/Cas9 system was first reported in 2013 [53,54,55]. In addition to the pioneer studies in the model plants *Arabidopsis* [56,57] and *Nicotiana* [53,54,58], the CRISPR/Cas9 system has been successfully used for genome editing in several fruit crops, including apple, banana, cacao, citrus, grape, kiwifruit, and pear. Several approaches have been evaluated to optimize the CRISPR/Cas9 technique for its use within a fruit cultivar. In most of these studies, the phytoene desaturase (*PDS*) gene, which encodes an enzyme in the carotenoid biosynthesis pathway was targeted. The disruption of this gene impairs chlorophyll and carotenoid production resulting in an albino phenotype [59] and is an easy target for manipulation to confirm the efficacy of the genome modification system. For instance, transgenic expression of Cas9 guided by 19-bp sgRNA designed to target the conserved region of two PDS genes in the banana genome resulted in complete albino and variegated phenotype among regenerated plantlets and mutation efficiency of 59% [60]. Higher editing efficiency (100%) targeting the same gene via polycistronic gRNAs was also described in banana [61]. Mutations induced in the *PDS* gene in diploid and octoploid strawberry resulted in a clear albino phenotype at a high frequency [62]. Similar results were seen following the editing of the *PDS* gene of Carrizo citrange. When the *Cas9* gene was driven by the *Arabidopsis YAO* gene promoter instead of the commonly used cauliflower mosaic virus 35S promoter, the mutation efficiency increased and in several instances was reported to be 100% [63]. Mutations in the *PDS* gene introduced by CRISPR/Cas9 have also been shown to confer an albino phenotype in apple [64,65], grapes [66] kiwifruit [67], pear [64], and kumquat [68].

The long juvenile phase observed in many perennial fruit crops results in an extended non-flowering period. This can last from 3 to 15 years depending on the fruit crop. Such a long juvenile phase hampers cultivars’ development efforts through traditional breeding [69,70]. Juvenility is usually associated with high levels of terminal flowering (*TFL*) protein [71]. *TFL* acts as a negative regulator of flowering by inhibiting the expression of several proteins that stimulate flowering, such as the FLOWERING LOCUS T (*FT*), LEAFY (*LFY*) and APETALA1 (*AP1*) [71,72]. Utilizing the CRISPR/Cas9 system, Charrier et al. [64] choose to edit the apple *TFL1*. They used two different sgRNAs targeting the *TFL1* gene. Additionally, the same construct was used to edit the pear TFL1, despite the presence of one mismatch between the sgRNA1 and the target. Early flowering was observed in 93% of the apple transgenic lines targeted in *MdTFL1.1* gene and 9% of the pear transgenic lines targeted in *PcTFL1.1*. The presence of this mismatch could explain the lower rate of the mutated phenotype observed in pear. It is also possible, that in pear, the editing of both TFL1 genes (*PcTFL1.1* and *PcTFL1.2*) is necessary to completely release the floral repression. In a different strategy, the CRISPR/Cas9 system was used to simultaneously target two kiwifruit CEN-like genes *AcCEN4* and *AcCEN*. Mutations in these genes transformed a climbing woody perennial, which develops axillary inflorescences after many years of juvenility, into a compact plant with rapid terminal flower and fruit development [73].

Fruit crops are subject to numerous pests and diseases, caused by fungi, bacteria, nematodes, and viruses. These factors hinder plant growth and development, and directly affect yield. The economic importance of each varies from time and region, but generally increases costs production and may lead to significant losses. The development of resistant/tolerant cultivars is among the most effective and economical alternatives to solve these problems. CRISPR/Cas9-mediated resistance to these biotic stresses can thus have a huge impact on their production. *Citrus sinensis LATERAL ORGAN BOUNDARIES* (*CsLOB1*) is a susceptibility gene for citrus canker, a disease caused by the bacterial pathogen *Xanthomonas citri* subspecies *citri* (*Xcc*) [74,75]. This gene has specific elements in the promoter region to which the *Xcc* pathogenicity factor *PthA4* binds, leading to *CsLOB1* induction. It was observed that by modifying the *PthA4* effector *cis*-elements in the promoter of *CsLOB1* by using the CRISPR/Cas9 system there was a reduced *Xcc* infection on the mutant plants [76]. These results revealed though that only plants with mutations in the promoters of both alleles of *CsLOB1* were resistant to citrus canker, suggesting that activation of a single allele of the *CsLOB1* gene via the *PthA4* binding element is sufficient for disease initiation. Similarly, Peng et al. [77] designed five CRISPR/Cas9 constructs to modify the *PthA4* binding element in the *CsLOB1* promoter of “Wanjincheng” orange. The mutation rate ranged from 11.5–64.7% depending on the construct used, and four mutated lines had enhanced resistance to citrus canker. Deletion of the entire *PthA4* binding element from both *LOB1* alleles resulted in significant citrus canker tolerance [77]. In grapes, CRISPR/Cas9-mediated knockout of *WRKY52*, encoding a transcription factor related to biotic stress responses, enhanced resistance to *Botrytis cinerea* [78]. Four gRNAs were designed for different regions in the first exon of *WRKY52* to guide the Cas9 nuclease. It was observed that transgenic biallelic mutant lines were more resistant than monoallelic mutant lines. In cacao, CRISPR/Cas9 was used to edit the *NON-EXPRESSOR OF PATHOGENESIS-RELATED GENES3* (*NPR3*) gene, which encodes a defense response repressor [79]. Gene editing was first tested by transient expression of the CRISPR/Cas9 cassettes in cacao leaf tissues, leading to deletions in 27% of the *NPR3* copies in the treated tissues and *Phytophthora tropicalis* resistance in the edited tissues. The authors hypothesized that cells in which *NPR3* was mutated activated their defense responses. Subsequently, they generated stably transformed and genome-edited somatic embryos for future testing the effectiveness of CRISPR/Cas9 at a whole plant level in cacao [80]. Banana streak virus (BSV) is one of the most important limitations for banana production. The dsDNA virus integrates into the host’s genome, producing infectious viral particles under stress conditions [81]. The CRISPR/Cas9 system was utilized to generate mutations in the BSV sequences integrated into the host genome of plantain cultivar Gonja manjaya. Seventy-five percent (6 out of 8) of regenerated genome-edited events tested remained asymptomatic in comparison to the non-edited plants under water stress conditions [82].

Recently, CRISPR/Cas9 was also utilized to induce mutations in the *MaGA20ox2* gene. This gene regulates dwarfism in banana [83]. Seven mutant lines were detected with semi—dwarf phenotype following genome editing. In this study, endogenous GA levels in the different tissues were quantified and the results were consistent with the phenotype observed. There was a significant change in GAs levels in the mutants compared to the non-transformed control in both leaves and roots [84]. This strategy can be subsequently utilized to develop the much-needed semi—dwarf or dwarf banana cultivars. Other crops that have been modified using the CRISPR/Cas9 system are outlined in Table 1.

## 4. Genome Editing in Vegetable Crops

As with the fruit crops, vegetables are also susceptible to a plethora of abiotic and biotic stresses that can make optimal production challenging, which emphasizes the importance of developing resistant/tolerant cultivars [95]. Additional areas of improvement in many vegetables include flavor and nutritional profile, plant architecture, and shelf life [96,97,98]. The CRISPR/Cas9 technology has been utilized to edit the genome of several commercially important vegetables to achieve these and other goals as outlined in Table 2.

Mainly utilized as a proof of concept, the CRISPR/Cas9 system has been used to induce mutations in the *PDS* gene in several vegetable crops such as cabbage, Chinese kale, tomato, and watermelon [101,108,127,132]. However, there is no economic benefit and these studies can only report on the effectiveness of the genome modification system on a specific crop. Among vegetables, it can be said that tomatoes are the species that gathers the largest number of studies with CRISPR/Cas9 system, either because of the economic importance of the crop or the ease of genetic transformation using *Agrobacterium.* In tomatoes, parthenocarpy is potentially a desirable trait because of consumers’ preference and for processing purposes [133]. The CRISPR/Cas9 system can be used as a breeding tool for the development of parthenocarpic tomato plants [116]. Initially, five different sgRNAs were tested on the tomato variety Micro-Tom to introduce mutations into SlIAA9, a key gene that controls parthenocarpy. The sgRNA2 induced the highest mutation efficiency and was subsequently used to transform the commercial cultivar Ailsa Craig. The authors also obtained bi-allelic and homozygous mutations in T0 regenerated plants of both Micro-Tom and Ailsa Craig. As expected, regenerated mutants exhibited the production of seedless fruit. Similarly, following the modification of the SlAGAMOUS-LIKE 6 (*SlAGL6*) gene in tomato, plants with a homozygous or biallelic mutation in the *SlAGL6* gene produced mostly parthenocarpic fruits and some low-seeded fruits (containing up to 10 seeds) [119].

Improving quality traits to make a vegetable more nutritious with a longer shelf life has been a key objective in many plant breeding programs, especially for fleshy fruit and vegetables. Post-harvest losses are an enduring threat in the production chain and result in decreased returns and lowers profits [134]. To develop tomatoes with a long shelf life the allele of *Alcobaca* (*SlALC*) was replaced by recessive alc via the HDR repair pathway. Subsequently, it was possible to obtain an *alc* homozygous mutants free of exogenous DNA with superior storage performance compared to wild type controls [124]. Other traits that have been successfully modified to improve the tomato include lycopene enhancement and fruit quality traits. Genome editing of several genes in the carotenoid metabolic pathway was conducted to promote lycopene biosynthesis, and at the same time inhibit its conversion to β- and α-carotene [118]. The lycopene content was increased by about 5.1-fold in several edited plants.

γ–aminobutyric acid (GABA) is regarded as a health-promoting functional compound and has received much attention in classical tomato breeding studies [135]. Editing several genes in the GABA pathway resulting in a 19—fold higher increase in the GABA content in tomato genome-edited plants [127]. In potato, reducing steroidal glycoalkaloids (SGAs) such as α-solanine and α-chaconine content in tubers is a key requisite for breeding superior potatoes, since the presence at high levels may confer a bitter taste and potential undesirable effects on humans. Thus, the CRISPR/Cas9 system was used to target a steroid 16α-hydroxylase (*St16DOX*) in SGA biosynthesis in potato. Two SGA-free potato lines carrying some deletions in the *St16DOX* gene were generated in this study [112].

To hasten the maturity and reduce the duration it takes for the tomato fruit to ripen, APETALA2a (*AP2a*), NON-RIPENING (*NOR*), and FRUITFULL (*FUL1/TDR4* and *FUL2/MBP7*) were edited [136]. Edited plants were early ripening in nature and this study also provided new insights into the role of *FUL1* and *FUL2* during fruit ripening. Several interesting features like day-neutral, enhanced compact determinate growth, accelerated flower production and early yield was also observed in *SELF PRUNING 5G*-edited CRISPR/Cas9 tomato plants [121].

Weed infestation causes serious problems during vegetable production and its control using selective herbicides is an important form of management. Herbicide-resistant watermelon plants were produced by generating single point mutation through CRISPR/Cas9 at a conserved position of acetolactate synthase (*ALS*) gene, a key enzyme for biosynthesis of branched-chain amino acids, valine, leucine, and isoleucine [137]. Similarly, the *acetolactate synthase* (*ALS*) gene in tomato and potato was also edited to induce herbicide resistance. Cytidine base editing (CBEs) techniques that results in a C-to-T base conversion was used. As a result chlorsulfuron-resistant plants were obtained with a precise base editing efficiency of up to 71% in tomato. More importantly 12.9% and 10% transgene-free edited plants were produced in the first generation in tomato and potato respectively [131]. Other traits that were modified include the enhancement of drought tolerance. The *SlNPR1,* which is a master regulator involved in plant defense response to pathogens was modified [130]. *slnpr1* mutants exhibited reduced drought tolerance with increased stomatal aperture, higher electrolytic leakage, malondialdehyde (MDA), and hydrogen peroxide (H_2_O_2_) levels, and lower activity levels of antioxidant enzymes, compared to the wild type (WT) plants.

Disease resistance through genome editing is also an emerging area of research in vegetables and is beginning to attract attention now that the CRISPR/Cas system has become regular for the genome editing of several plant species. A tomato plant resistant to powdery mildew disease was generated by utilizing two sgRNAs that introduced specific mutations (48-bp deletion in a homozygous configuration) in the *MLO1* locus, the major contributor to susceptibility to the fungal pathogen *Oidium neolycopersici* [123]. Broad-spectrum bacterial disease resistant tomato plants were also obtained by editing the tomato *SlDMR6-1* (downy mildew resistance 6 gene) ortholog [138]. Also, by disrupting the recessive *eIF4E* (eukaryotic translation initiation factor 4E) gene, virus-resistant cucumber plants could be obtained [107]. As more and more targets are identified, it is anticipated that there will be several more successful studies on heritable disease resistance using the CRISPR/Cas system in a wider range of vegetable crops.

## 5. Genome Editing in Ornamental Crops

Unlike fruits and vegetables which are mainly grown for human consumption, ornamentals are grown for their aesthetic values. CRISPR/Cas9 genome editing technology can be therefore utilized to improve the plant architecture, modify the color, fragrance, size, and shelf life of the flowers. Additionally enhanced abiotic and biotic stress resistance is always desirable for any crop cultivar [139,140,141,142]. Genetic transformation of ornamentals enables the production of high quality flowers and generate plants with novel colors and shapes. However, just like fruits and vegetables, there are some ornamental cultivars that are difficult to transform using *Agrobacterium*. In these cases, a proof of concept study is usually necessary before devising plans to edit for useful traits for the improvement of the cultivar. In most of these studies, the *PDS* gene is targeted. The *Lilium LpPDS* gene was successfully edited in two *Lilium* species for the first time and completely albino, pale yellow, and albino–green chimeric mutants were observed [143]. Transforming petunias with a CRISPR/Cas9 construct targeting *PDS* also result in albino phenotype [144].

Flower initiation and development is a major stage in the ornamental plant’s life cycle. Utilizing newer approaches such as the CRISPR/Cas9 to improve on the flower characteristics has distinct economic advantages [145]. CRISPR/Cas9 techniques were used to target multiple *MADS* genes in the *Phalaenopsis* orchid. Several MADS—null mutants were generated [146]. *MADS* genes are highly expressed in floral organs and may be important for flower initiation and development [147] and it remains to be seen how these *Phalaenopsis* lines will perform once they become mature. Flower longevity is another important characteristic of all ornamental plants. Many of them suffer from a reduction in flower longevity primarily due to enhanced ethylene production [148]. Several chemicals have been utilized to enhance longevity but these cannot fully stop the senescence process [149]. Ethylene production can be decreased by targeting a key enzyme in the ethylene biosynthesis pathway (1-aminocyclopropane-1-carboxylate oxidase [*ACO*]) [150]. When the petunia *PhACO1* was edited using CRISPR/Cas9, mutant lines improved flower longevity compared with the wild-type [151]. Genes responsible for other flower traits have also been edited. When the carotenoid cleavage dioxygenase (*CCD*) was edited in *Ipomoea nil*, it resulted in a 20-fold increased carotenoid content in the petals of the CRISPR edited plants [152]. Mutations in the *Torenia fournieri* flavanone 3-hydroxylase (F3H) gene resulted in pale blue flowers at a high frequency (ca. 80% of regenerated lines) in edited torenia plants [153]. Additionally, several other ornamental cultivars have been successfully edited using the CRISPR/Cas9 system and are listed in Table 3.

## 6. CRISPR/Cas9 Genome Editing for Generation of Non-Transgenic Horticultural Crops

Significant efforts have been made to improve yield, increase abiotic and biotic resistance, or enhance quality to satisfy the ever-changing consumer’s requirements. Historically, most of the successful outcomes have been accomplished through conventional breeding. A majority of the horticultural crops, however, exhibit either high levels of heterozygosity, self- and cross-incompatibility, long juvenile period and complex genome (triploid or polyploid species) or a combination of them [157,158]. During the last 20 years, genetic engineering has appeared as a useful technique to supplement crop breeding for those species, although it also has its limitations [159,160]. In transgenic plants, “foreign DNA” including selectable marker genes is introduced. The integration of the exogenous DNA in a random fashion may also interrupt endogenous genes or modify the expression of neighboring genes [161,162]. One major advantage of CRISPR/Cas9 over transgenic approaches is the possibility of generating non-transgenic plants. Since the CRISPR/Cas9 expression cassettes and their target sites are at different locations in the genome, segregation and removal of the CRISPR/Cas9 cassettes are possible through subsequent generations of selfing or crossing. Edited tomato plants could be generated containing the desired mutations and phenotype (resistant to powdery mildew disease) but transgene-free [116]. To introduce mutations, two sgRNAs were introduced in the tomato and which was removed through gene segregation in the subsequent generations.

However, segregation and removal of the CRISPR/Cas9 cassettes through selfing or crossing is not feasible in most of the fruits trees since they have a long juvenile stage, requiring several years to reach the reproductive stage. Besides, many of them have a complex genome with a high level of heterozygosity and polyploidy and are usually vegetatively propagated. In such cases, transgene-free edited plants can be generated by transiently expressing the CRISPR/Cas9 components in the nucleus. This is feasible since the unintegrated CRISPR/Cas9 cassettes can still be expressed and function for a short time necessary for introducing precise mutations. The feasibility of this method was demonstrated on apples with the production of T-DNA free edited apple lines [64]. However, the overall efficiency of this transient system was very low (0.4% of edited lines), but it is only a matter of time before advances in the genetic transformation process results in higher efficiency to make this a routine editing process.

Delivering preassembled Cas9 protein—gRNA ribonucleoproteins (RNPs) into plant cells can also result in genome editing without transgene integration. The RNPs directly edit the target sites after delivery in cells and are subsequently degraded by endogenous proteases. Thus, there is no DNA integration in the genome [85,126]. In order to adopt a DNA-free delivery protocol for grapevine and apple, Malnoy et al. [85] transformed protoplasts with purified Cas9 ribonucleoproteins while Woo et al. [126] transformed lettuce protoplasts in a similar manner. Mutagenesis efficiencies of between 0.1% and 0.5–6.9% were achieved in grapevine and apple using this system. However, no plants were regenerated. Osakabe et al. [91] also developed a stepwise protocol for the design and transfer of CRISPR–Cas9 components with high accuracy and efficiency in apple and grapevine protoplasts. The complete protocol employing the direct delivery of CRISPR–Cas9 RNPs takes as little as 2–3 weeks, with the advantage of generating exogenous DNA-free plants, whereas the plasmid-mediated procedure takes more than 3 months to regenerate plants and study the mutations.

Generation of transgene-free plants using the CRISPR/Cas9 technology is important given the current stringent and costly regulations on genetic modification. Although the regulation on gene-edited crops is still being debated, plants that are free of transgenes may not be subjected to the existing regulations on genetic modification [163,164], possibly reducing the investment in time and money. Private companies, for example, have invested in the development of transgenic crops, such as soybeans and maize, which have brought great financial returns. This investment is obviously an obstacle for the development of transgenic fruit crops, which are mostly perennial and vegetatively propagated. Thus, the prospect of facilitating the commercial release for CRISPR-plants free of transgenes could benefit not only private companies but also public research institutions encouraging them to invest in the development of transgene-free gene-edited fruit crops. In addition, it can facilitate public acceptance since it is a plant that is not considered transgenic. From all this, we can assume that transgene-free genome editing methods have the potential to be a powerful tool for genetic improvement of many fruit crops.

## 7. Challenges of CRISPR/Cas9 Genome Editing

The CRISPR/Cas9 system is a promising, revolutionary technology for crop breeding and biological research through directed and controlled changes in the genome. To date, most CRISPR/Cas9 studies in horticultural crops involve NHEJ-mediated gene repair to create precise mutations to knock out or modify the target gene’s function. In many cases, the resulting phenotype confirmed the effectiveness of the technique or revealed a specific gene function and, in others, desirable agronomic traits such as enhancement of disease resistance was created (Table 1, Table 2 and Table 3). These results make it clear that the CRISPR/Cas9 system is highly valuable in horticultural crop-specific applications. However, there are still some obstacles to be overcome. First, in order to design specific sgRNA and prevent off-target gene editing, the target organism must have its genome sequenced. It is difficult to conduct gene-editing work in organisms without whole-genome sequence. High-quality genome assemblies have been developed for species such as banana, peach, raspberry, cocoa, papaya, clementine mandarin, coffee, and grape while the genome sequences of other popular species such as olive and avocado, as well as tropical crops, are not yet available or the quality can be improved [165]. The availability of newer sequencing platforms may facilitate cost-effective and in-depth sequencing of the entire genome of all commercially important horticultural species, as well as result in a better understanding of their genome structure, gene pathways, and gene function making CRISPR/Cas9 more useful for breeding of those crops.

Polyploidy is a widespread feature in plant genomes, including many horticultural crop species. For example, commercial varieties of kiwifruit, strawberry, and banana are polyploid which complicates both breeding and genome editing. To reach the expected phenotype it is necessary to mutate two or more copies of the target gene. Thus, a highly efficient editing platform for creating biallelic or multiallelic mutations within the same generation is essential for the future improvement of these and other crops [61,62,67]. Target site selection and sgRNA design, application of multiple gRNAs for the same target, suitable promoter to express both the gRNA and Cas9 are some important factors that must be addressed to ensure a higher frequency of induced mutations [166,167,168].

Another basic requirement is an efficient method for introducing or transiently expressing the CRISPR/Cas9 components into plant cells and subsequently in vitro generation of complete plants from these modified cells. Plant transformation technologies have been developed in most commercially important horticultural crops during the last 30 years [169]. This indicates that there are standardized transformation and regeneration protocols in many crops. However, genetic transformation methods including *Agrobacterium*-mediated or transformation with purified ribonucleoproteins into protoplasts are typically genotype-specific and require optimization of multiple parameters for its use within a species to achieve high efficiency. Therefore, the development of efficient and reproducible delivery systems, as well as selection and regeneration protocols, would be of critical importance for bringing CRISPR/Cas9 for routine use in horticultural crops.

The new gene editing techniques are more precise than standard genetic engineering tools that have been previously developed. There has been some concerns about the effect of mutations on non-target genes (“off-target”), possibly leading to unintended effects, which can happen especially for species with large and complex genomes [170]. However, gene editing can also lead to unintended effects even when mutations occurs “on-target.” Small insertion or deletion of DNA usually modify the reading code of the gene, preventing protein production or even producing altered proteins, with an unknown effect. These undesirable mutations should be identified prior to cultivar use, although procedures for the detection (bioinformatics and next generation sequencing) still face potential shortfalls. Even though there are questions about the risk of genome editing in plants it is important to understand that CRISPR/Cas9 is a relatively recent technique and the current knowledge about its safety is limited [171].

## 8. Conclusions

The CRISPR/Cas9 system is a promising, revolutionary technology for crop breeding and biological research through directed and controlled changes in the genome. Several successful examples have already been achieved in many important horticultural crops. The expanding knowledge on CRISPR/Cas9-based tools, especially strategies that allow the development of a non-transgenic plant, delivery methods and genomic information will lead to the development of horticultural crops with improved agronomic traits, bringing about innovative solutions for sustainable and competitive food production.

## Figures and Tables

**Table 1:** List of targeted gene(s) and trait modified via the CRISPR/Cas9 system in different fruit crops.

Species	Target Gene	Target Trait	Delivery Method	Reference
Apple	DIPM-1, DIPM-2, DIPM-4	Fire blight disease resistance	PEG-mediated protoplast transfection	[85]
Apple	PDS	Albino phenotypes	*Agrobacterium*-mediated leaf discs transformation	[65]
Apple and Pear	PDS	Albino phenotypes	vacuum-infiltration in a suspension of *Agrobacterium tumefaciens*	[64]
	TFL1	Early flowering		
Banana	PDS	Albino phenotype	*Agrobacterium*-mediated suspension cells transformation	[61]
Banana	PDS	Albino phenotypes	*Agrobacterium*- mediated embryogenic cell suspension cultures transformation	[60]
Banana	MaGA20ox2	semi—dwarfing size	*Agrobacterium*-mediated suspension cells transformation	[84]
Banana	eBSV	Control of virus pathogenesis	*Agrobacterium*-mediated suspension cells transformation	[82]
Cacao	TcNPR3	Phytophthora tropicalis resistance	*Agrobacterium*-mediated transient leaf transformation	[80]
Citrus (Carrizo Citrange)	PDS	Albino phenotypes	*Agrobacterium*-mediated epicotyl transformation	[63]
Citrus (Grapefruit)	CsLOB1	Canker disease resistance	*Agrobacterium*-mediated epicotyl transformation	[86]
Citrus (Grapefruit)	PDS	Albino phenotype	*Agrobacterium*-mediated epicotyl transformation	[87]
Citrus (Kumquat)	PDS	Albino phenotypes	*Agrobacterium*-mediated epicotyl transformation	[68]
Citrus (Sweet Orange)	CsLOB1	Canker disease resistance	*Agrobacterium*-mediated epicotyl transformation	[77]
Citrus (Sweet Orange)	CsWRKY22	Canker disease resistance	*Agrobacterium*-mediated epicotyl transformation	[88]
Citrus (Sweet Orange)	DMR6	Huanglongbing resistance	*Agrobacterium*-mediated epicotyl transformation	[89]
Grape	PDS	Albino phenotypes	*Agrobacterium*- mediated callus transformation	[66]
Grape	PDS	Albino phenotype	*Agrobacterium*-mediated suspension cells transformation	[90]
Grape and Apple	IdnDH	Biosynthesis of tartaric acid	*Agrobacterium*-mediated suspension cells transformation	[91]
Grape	L-idonate dehydrogenase gene (IdnDH)	Tartaric acid content	*Agrobacterium*-mediated suspension cells transformation	[92]
Grape	VvWRKY52	Botrytis cinerea resistance	*Agrobacterium*-mediated somatic embryos transformation	[78]
Kiwifruit	PDS	Albino phenotype	*Agrobacterium*-mediated transformation	[67]
Strawberry	APETALA3 (AP3)	Flowering control	*Agrobacterium*-mediated leaf disk	[93]
Strawberry	Auxin Response Factor 8 (FvARF8) and Auxin biosynthesis gene (FveTAA1)	Auxin biosynthesis	*Agrobacterium*-mediated transformation	[94]
Strawberry	PDS	Albino phenotypes	*Agrobacterium*-mediated leaf and petiole transformation	[62]

**Table 2:** List of targeted gene(s) and trait modified via the CRISPR/Cas9 system in different vegetable crops.

Species	Target Gene	Target Trait	Delivery Method	Reference
*Brassica campestris*	pectin-methylesterase genes *Bra003491*, *Bra007665*, and *Bra014410*	methylation of pectin	*Agrobacterium*-mediated transformation	[99]
*Brassica oleracea* var. capitata	Phytoene desaturase gene *BoPDS*, the S-receptor kinase gene *BoSRK*, and the male-sterility-associated gene *BoMS1*	Albino phenotypes, Male sterility, self-incompatibility	*Agrobacterium*-mediated transformation	[100]
Cabbage	*BoPDS*	Albino phenotypes	*Agrobacterium*-mediated hypocotyl transformation	[101]
Cabbage	*PDS* and *FRI*	Albino phenotype and flowering	PEG-mediated protoplast transfection	[102]
Red cabbage	centromere-specific histone H3 (*CENH3*)	haploid lines induction	protoplast transformation and Agro infiltration	[103]
Carrot	Flavanone-3-hydroxylase (*DcF3H*)	Anthocyanin biosynthesis blockage	*Agrobacterium*-mediated callus transformation	[104]
Carrot	*DcMYB113-like*	Anthocyanin biosynthesis	*Agrobacterium*-mediated transformation	[105]
Chicory	*CiPDS*	Albino phenotype	*Agrobacterium*-mediated leaf sections and protoplast transfection	[106]
Cucumber	Eukaryotic translation initiation factor 4E (*eIF4E*)	Virus resistance	*Agrobacterium*-mediated cotyledon transformation	[107]
Kale	*PDS*	Albino phenotypes	*Agrobacterium*-mediated transformation	[108]
Lettuce	*LsBIN2*	Impaired brassinosteroid response	PEG-mediated protoplast transfection	[109]
Lettuce	*LsNCED4*	Thermo-inhibition of seed germination	*Agrobacterium*-mediated cotyledon segments transformation	[110]
Potato	Acetolactate synthase1 (*StALS1*)	Herbicide resistance	*Agrobacterium*-mediated leaf transformation	[111]
Potato	16α-hydroxylation (*St16DOX*)	Steroidal glycoalkaloids (SGAs) biosynthesis	*Agrobacterium*- mediated shoots transformation	[112]
Potato	Granule-bound starch synthase (*StGBSS*)	Starch quality	PEG-mediated protoplast transfection	[113]
Potato	*StIAA2*	Aux/IAA protein	*Agrobacterium*- mediated stem segments transformation	[114]
Potato	*SBE1*, *SBE2*	Starch quality	PEG-mediated protoplast transfection	[115]
Tomato *	Aux/IAA9 (*SlIAA9*)	Parthenocarpic Fruits	*Agrobacterium*-mediated leaf disk transformation	[116]
Tomato	Carotenoid cleavage dioxygenase 8	Resistance against *Phelipanche aegyptiaca*	*Agrobacterium*- mediated transformation	[117]
Tomato	*SGR1*, *lycopene ε-cyclase, beta-lycopene cyclase, lycopene β-cyclase1, and LCY-B2*	Lycopene content	*Agrobacterium*-mediated transformation	[118]
Tomato	SlAGAMOUS-LIKE 6 *(SlAGL6)*	Parthenocarpic Fruits	*Agrobacterium*-mediated transformation	[119]
Tomato	Ripening inhibitor (*RIN*)	MADS-box transcription factor regulating fruit ripening	*Agrobacterium*-mediated transformation	[120]
Tomato	Self-pruning 5G (*SlSP5G*)	Day-length-sensitive flowering	*Agrobacterium*-mediated transformation	[121]
Tomato	Blade-on-petiole (*SlBOP*)	Inflorescence architecture	*Agrobacterium*-mediated cotyledon segments transformation	[122]
Tomato	Mildew Resistant Locus 1 (*SlMlo1*)	Powdery mildew resistance	*Agrobacterium*-mediated transformation	[123]
Tomato	Alcobaca (*SLALC*)	Long-shelf Life	*Agrobacterium*-mediated hypocotyls transformation	[124]
Tomato	*lncRNA1459*	Fruit ripening repress	*Agrobacterium*-mediated transformation	[125]
Tomato	*PDS*	Albino phenotypes	*Agrobacterium*-mediated transformation	[126]
Tomato	*SlyPDS, SlyGABA* *–TP1, SlyGABA* *–TP2, SlyGABA* *–TP3, SlyCAT9, and SlySSADH*	Albino phenotype; γ–aminobutyric acid (GABA)	*Agrobacterium*-mediated transformation	[127]
Tomato	(Methyltransferase 1) *SlMET1*	DNA methylation	*Agrobacterium*-mediated transformation	[128]
Tomato	enzymes pectate lyase (*PL*), polygalacturonase 2a (*PG2a*), and β-galactanase (*TBG4*)	Pectin Degradation control	*Agrobacterium*-mediated transformation	[129]
Tomato	*NPR1*	drought tolerance	*Agrobacterium*-mediated cotyledon segments transformation	[130]
Tomato and potato	*SlALS2*	Herbicide resistance	*Agrobacterium*-mediated transformation	[131]
Watermelon	*PDS*	Albino phenotype	*Agrobacterium*-mediated callus transformation	[132]

* Not an exhaustive list.

**Table 3:** List of targeted gene(s) and trait modified via the CRISPR/Cas9 system in different ornamental crops.

Species	Target Gene	Target Trait	Delivery Method	References
*Chrysanthemum morifolium*	Yellowish-green fluorescent (*CpYGFP*)	Fluorescence protein disruption	*Agrobacterium*-mediated leaf sections *transformation*	[154]
*Ipomoea nil*	carotenoid cleavage dioxygenase (*CCD*)	carotenoid accumulation regulation	*Agrobacterium*-mediated immature embryo	[152]
*Lilium longiflorum, Lilium pumilum*	*LpPDS*	Albino phenotype	*Agrobacterium*-mediated callus transformation	[143]
Petunia	Phytoene desaturase (*PhPDS*)	Albino phenotype	*Agrobacterium*-mediated leaf discs transformation	[144]
Petunia	Nitrate reductase (*PhNR*)	Deficiency in nitrate assimilation	PEG-mediated protoplast transfection	[155]
Petunia	*PhACO* genes (*PhACO1*, *PhACO3*, and *PhACO4*)	flower longevity	PEG-mediated protoplast transfection	[151]
Petunia	*PiSSK1*	Self-incompatibility	*Agrobacterium*-mediated transformation	[156]
Phalaenopsis orchid	*MADS*	Floral initiation and development	*Agrobacterium*-mediated protocorms transformation	[146]
*Torenia fournieri*	flavanone 3-hydroxylase gene (*F3H*)	flavonoid biosynthesis	*Agrobacterium*-mediated leaf sections	[153]
